# Chemical Pattern Recognition for Quality Analysis of Lonicerae Japonicae Flos and Lonicerae Flos Based on Ultra-High Performance Liquid Chromatography and Anti-SARS-CoV2 Main Protease Activity

**DOI:** 10.3389/fphar.2021.810748

**Published:** 2022-01-04

**Authors:** Lifei Gu, Xueqing Xie, Bing Wang, Yibao Jin, Lijun Wang, Guo Yin, Jue Wang, Kaishun Bi, Tiejie Wang

**Affiliations:** ^1^ NMPA Key Laboratory for Quality Research and Evaluation of Traditional Chinese Medicine, Shenzhen Institute for Drug Control, Shenzhen, China; ^2^ Shenzhen Key Laboratory of Drug Quality Standard Research, Shenzhen Institute for Drug Control, Shenzhen, China; ^3^ School of Pharmacy, Shenyang Pharmaceutical University, Shenyang, China

**Keywords:** Lonicerae japonicae Flos, Lonicerae Flos, Anti-SARS-CoV2 activity, chemical pattern recognition, quality analysis

## Abstract

Lonicerae japonicae flos (L. japonicae flos, *Lonicera japonica* Thunb.) is one of the most commonly prescribed botanical drugs in the treatment or prevention of corona virus disease 2019. However, L. japonicae flos is often confused or adulterated with Lonicerae flos (L. flos, *Lonicera macrantha* (D.Don) Spreng., Shanyinhua in Chinese). The anti-SARS-CoV2 activity and related differentiation method of L. japonicae flos and L. flos have not been documented. In this study, we established a chemical pattern recognition model for quality analysis of L. japonicae flos and L. flos based on ultra-high performance liquid chromatography (UHPLC) and anti-SARS-CoV2 activity. Firstly, chemical data of 59 batches of L. japonicae flos and L. flos were obtained by UHPLC, and partial least squares-discriminant analysis was applied to extract the components that lead to classification. Next, anti-SARS-CoV2 activity was measured and bioactive components were acquired by spectrum-effect relationship analysis. Finally, characteristic components were explored by overlapping feature extracted components and bioactive components. Accordingly, eleven characteristic components were successfully selected, identified, quantified and could be recommended as quality control marker. In addition, chemical pattern recognition model based on these eleven components was established to effectively discriminate L. japonicae flos and L. flos. In sum, the demonstrated strategy provided effective and highly feasible tool for quality assessment of natural products, and offer reference for the quality standard setting.

## Introduction

The ongoing outbreak of the corona virus disease 2019 (COVID-19) infection has been a crisis of global health and economy (Hu et al., 2020; Rothan and Byrareddy, 2020; Wu et al., 2020). As of November 5, 2021, there are more than 248 million confirmed cases and 5.02 million related deaths caused by COVID-19 (WHO, 2021). Symptoms of COVID-19 are variable, but often include fever, cough, and breathing difficulties (Zhao et al., 2021). Since the COVID-19 pandemic, China’s treatment protocol using integrated traditional Chinese and Western medicine played significant efficacies in alleviating symptoms and suppressing the rapid spread of COVID-19 in China (Ni et al., 2020). Lonicerae japonicae flos (L. japonicae flos, *Lonicera japonica* Thunb.), a well-known heat-clearing and detoxifying botanical drug, is one of the most commonly prescribed botanical drugs in the treatment or prevention of COVID-19 according to an extensive analysis of the frequency of traditional Chinese medicine used in China (Luo et al., 2020). Clinical trials have confirmed that L. japonicae flos decoction efficiently inhibited severe acute respiratory syndrome coronavirus 2 (SARS-CoV2) replication and accelerated the negative conversion of infected patients (Zhou et al., 2020). Investigations have revealed that many Chinese medicinal prescriptions containing L. japonicae flos successfully relieved COVID-19 symptoms, improve the recovery of thoracic radiological abnormalities, and prevent fatal deterioration of the disease (Xiao et al., 2020; Hu et al., 2021; Huang et al., 2021).

Due to excellent anti-SARS-CoV2 activity, consumption of L. japonicae flos is increasing largely. However, L. japonicae flos is often confused or adulterated with Lonicerae flos (L. flos, *Lonicera macrantha* (D.Don) Spreng., Shanyinhua in Chinese) for they are two closely related botanical species with similar morphological characteristics and had been officially listed as a single item in the Chinese Pharmacopoeia (Zhang et al., 2015). Both of L. japonicae flos and L. flos possess heat-clearing and detoxifying efficacy (Li et al., 2019), while pharmacological investigation has revealed that the antibacterial intensity of L. japonicae flos was obviously different from L. flos (Shi et al., 2016). In addition, L. flos may have greater antioxidant activity than that of L. japonicae flos according to higher phenolic acids content in L. flos (Kong et al., 2017; Zuowei et al., 2019). Phytochemistry studies indicated that phenolic acids, flavonoids, iridoids, and saponins are the predominant composition of L. japonicae flos and L. flos, but saponins contentes in L. japonicae flos are fewer than those in L. flos (Li et al., 2019). On this account, L. flos is not allowed to prepare injections. Until now, there has been little work that completely investigated the anti-SARS-CoV2 activity and related chemical properties of L. japonicae flos and L. flos. Thus, for the sake of precise quality evaluation, the establishment of characterization methods for L. japonicae flos and L. flos according to their effectiveness is essential.

Chemical pattern recognition applies mathematics, statistics and computer science to analyze the chemical data, perform molecular feature extraction and establish classification models, which has been widely used in quality assessment of traditional Chinese medicine (Cao et al., 2018; Huang et al., 2020). Limited research has been performed for quality analysis of L. japonicae flos and L. flos based on anti-SARS-CoV2 activity using chemical pattern recognition method.

**FIGURE 1:**
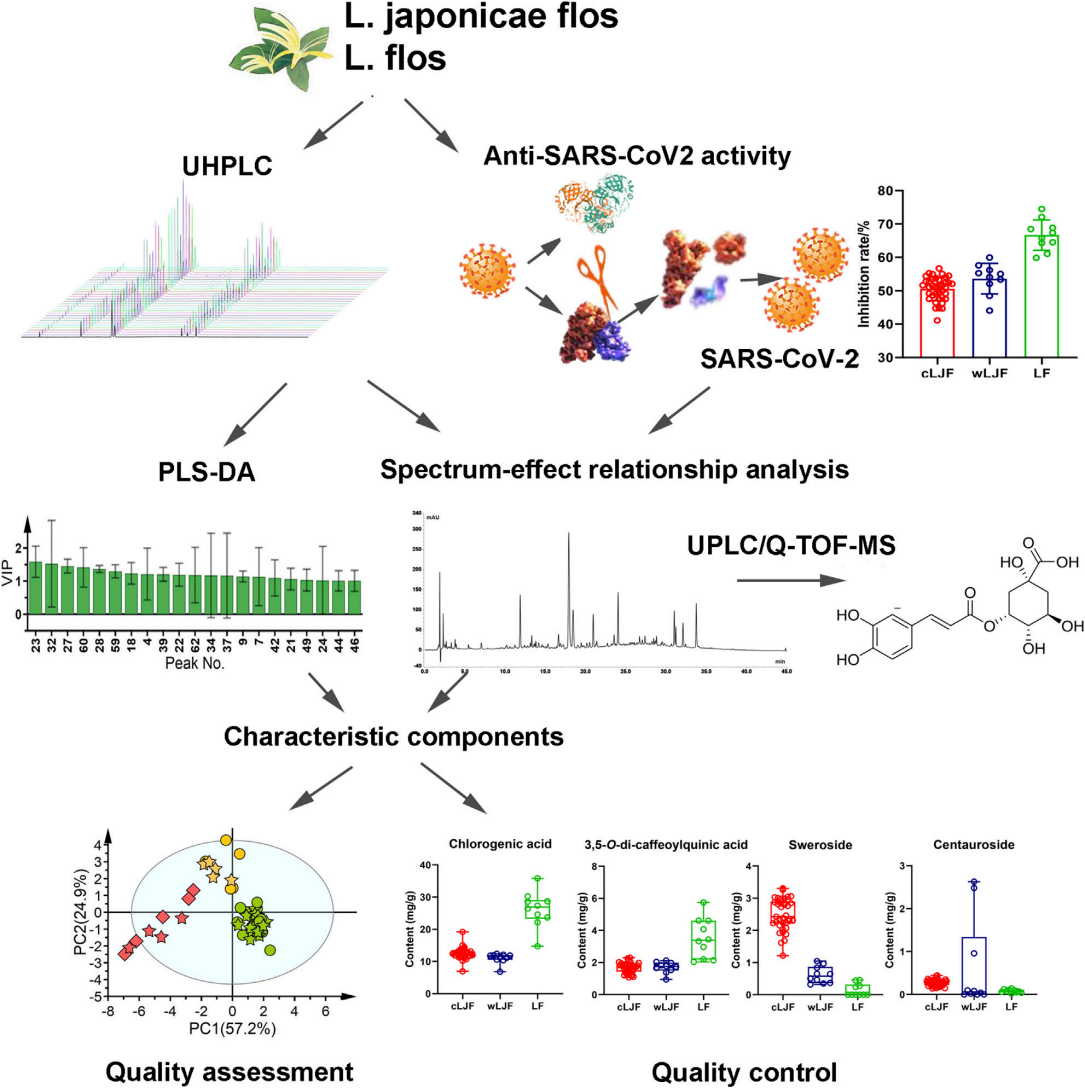
The strategy of exploring characteristic components and establishing quality assessment model of L. japonicae flos and L. flos based on anti-SARS-CoV2 activity.

In the present study, chromatographic profiles and anti-SARS-CoV2 activity data of L. japonicae flos and L. flos coupled with chemical pattern recognition method were integrated to discovery characteristic components and establish quality assessment model. Detailed strategy is summarized in Figure 1. First of all, chemical components that display critical role in classifying L. japonicae flos and L. flos were extracted by PLS-DA based on UHPLC profile data. Then, anti-SARS-CoV2 activity of 59 samples was measured to conduct spectrum-effect relationship analysis with chromatographic peaks data. Bioactive components were then identified by ultra-performance liquid chromatography coupled with quadrupole time-of-flight mass spectrometry (UPLC/Q-TOF-MS) analysis. Next, characteristic components were obtained by integrating feature extracted components and bioactive components. Chemical pattern recognition model was built to differentiate L. japonicae flos and L. flos based on characteristic components. In addition, characteristic components not only had important role in differentiating L. japonicae flos and L. flos samples, but also were closely related to anti-SARS-CoV2 activity, which could be recommend as quality control components.

## Materials and Methods

### Samples and Chemicals

To obtain anti-SARS-CoV2 activity and chemical diversity, 59 batches of dried L. japonicae flos (*Lonicera japonica* Thunb.) and L. flos (*Lonicera macrantha* (D.Don) Spreng.) samples were collected from China in May 2020, including 39 batches of cultivated L. japonicae flos, 10 batches of wild L. japonicae flos, and 10 batches of L. flos. The detailed sample information was listed in Supplementary Table S1 and the representative appearance was shown in Supplementary Figure S1. All samples were authenticated by Professor Ji Zhang at Beijing University of Chinese Medicine. Voucher specimens were deposited in the cold sample room, Shenzhen Institute for Drug Control.

Chlorogenic acid, 3,5-Di-*O*-caffeoylquinic acid, 4,5-Di-*O*-caffeoylquinic acid, morroniside, swertiamarin, and sweroside analytical standards were obtained from National Institutes for Food and Drug Control (Beijing, China) with certified purity of 98.3, 94.3, 94.1, 96.8, 98.3 and 98.3%, in order. Neochlorogenic acid, cryptochlorogenic acid, 3,4-Di-*O*-caffeoylquinic acid and secoxyloganin analytical standard were acquired from Shanghai Standard Technology Co., Ltd. (Shanghai, China) with certified purity of ≥ 98.0%. Centauroside was obtained from Sichuan Weikeqi Biological Technology Co., Ltd. (Chengdu, Sichuan, China) with certified purity of ≥ 98.0%. Acetonitrile of chromatographic grade was purchased from Merck KGaA (Darmstadt, Germany). Formic acid of chromatographic grade was purchased from Shanghai Aladdin Biochemical Technology Co., Ltd. (Shanghai, China). Milli-Q^®^ water was prepared in-house using a Milli-Q Academic ultra-pure water system (Millipore, Milford, MA, United States). Other chemicals used were of analytical grade. SARS-CoV2 M^pro^/3CL^pro^ Assay Kit was purchased from Nanjing Jiancheng Biotech Co., Ltd (Nanjing, Jiangsu, China).

### Preparation of Extracts, Sample Solutions and Analytical Standards

All dried L. japonicae flos and L. flos samples were accurately weighed at 6.00 g, soaked in 25-fold volumes of water (*w/v*) for 1 h, and boiled three times for 1 h each time, respectively. The aqueous extractions were concentrated under reduced pressure and lyophilized to powder. For experiment on anti-SARS-CoV2 activity, L. japonicae flos and L. flos extracts were also dissolved in water solution at required concentration, respectively. For ultra-high performance liquid chromatography (UHPLC) analysis, an aliquot of 100 mg of powdered sample, accurately weighed, was dissolved in water to a 10 ml volumetric flask. The sample aqueous solution was filtered through a 0.22 μm syringe filter and the successive filtrate was used as the test solution stored at 4°C before analyzing.

Analytical standards including chlorogenic acid, cryptochlorogenic acid, neochlorogenic acid, 4,5-*O*-dicaffeoyl quinic acid, 3,5-*O*-dicaffeoyl quinic acid, morroniside, 3,4-*O*-dicaffeoyl quinic acid, swertiamarin, sweroside, centauroside, and secoxyloganin were weighed accurately and dissolved with 50% methanol as the stock liquid, giving a concentration of 760.00 μg mL^−1^, 250.00 μg mL^−1^, 240.00 μg mL^−1^, 230.00 μg mL^−1^, 120.00 μg mL^−1^, 55.00 μg mL^−1^, 160.00 μg mL^−1^, 90.00 μg mL^−1^, 250.00 μg mL^−1^, 50.00 μg mL^−1^, and 270.00 μg mL^−1^, respectively.

### UHPLC Apparatus and Conditions

Chromatographic analyses were performed on a Thermo Scientific Ultimate 3,000 Series UHPLC system (Thermo Fisher Scientific, Bremen, Germany) equipped with a pump, an auto-sampler, a rapid separation-column compartment and a diode array detector. Chromatographic separation was carried on an Agilent Poroshell SB-C18 reverse-phase column (4.6 mm × 150 mm, 2.7 μm, Agilent Technologies Inc., Santa Clara, CA, United States). The column was eluted with a gradient concentration of acetonitrile and 0.1% formic acid aqueous solution at a flow rate of 0.9 ml/ min using the following program. Solvent A: 0.1% formic acid in Milli-Q^®^ water, solvent B: acetonitrile, 0–5 min, 5% B; 5–10 min, 5–10% B; 10–15 min, 10% B; 15– min, 10–20% B; 25–40 min, 20–30% B; 40–45 min, 30–40% B. The injection volume was set at 5 μL, 240 nm was chosen as the detection wavelength and the column temperature was maintained at 15°C.

### Mathematical Approaches for Molecular Feature Extraction

Partial least squares-discriminant analysis (PLS-DA) is a supervised pattern recognition method with priori information of the datasets. It has been widely used to perform feature extraction of chemical components by statistical software (Simca 14.0; Umetrics, Andover, MA, United States). In PLS-DA, the profile of variable importance in projection (VIP) could reflect the contribution levels of the main markers of categories differentiation. The values of VIP were greater than 1 and were mainly responsible for sample discrimination.

### SARS-CoV2 Main Protease (M^pro^) Activity Assay

All L. japonicae flos and L. flos samples were dissolved in phosphate buffer saline. The inhibition rate and half-maximal inhibitory concentration (IC_50_) were determined using SARS-CoV2 M^pro^/3CL^pro^ Assay Kit. In brief, different samples and SARS-CoV2 M^pro^ were pre-added to 96-well black flat-bottomed plates with a total volume of 98 μL. Afterward, a 2 μL fluorescence resonance energy transfer (FRET) substrate solution, Dabcyl-KTSAVLQSGFRKME-Edans (Xiao et al., 2021), was quick added to each well to initiate the reaction. After incubating at 37°C for 5 min in the dark, the fluorescence intensity (excitation/emission, 340 nm/490 nm) of released Edans were measured using a Thermo Scientific Microplate Reader (Thermo Fisher Scientific, Waltham, Mass, United States).

### Grey Relational Analysis

The grey relational analysis, a multi-factor statistical analysis method, was applied to rank the components which contributed to the anti-SARS-CoV2 M^pro^ activity. The chromatographic peak areas of various components in L. japonicae flos and L. flos samples (S1∼S59) were set as the characteristic sequence, while the corresponding inhibition rates to SARS-CoV2 M^pro^ were set as the compared sequence. In order to eliminate the adverse effects of inconsistent units of the two datasets, dimensionless processing was carried out on the original data. Specifically, each data were divided by average value of the corresponding sequence, and the resultant data were employed to calculate the relevancy degree. The grey relational degree between characteristic sequence and compared sequence was calculated with resolution ratio of 0.5 (Shi et al., 2020).

### Chemical Pattern Recognition Analysis

All fingerprint peak areas of UHPLC chromatograms were normalized by Z-score transformation method by Chromatographic Fingerprint of Traditional Chinese Medicine (Version 2004A, Chinese Pharmacopoeia Committee). A total of 59 batches of samples were divided into three sets, including training set (S1∼S5, S14∼18, S27∼S31, S37∼S44, S50∼S54), testing set (S6∼S11, S13, S19∼S23, S26, S32∼S36, S45∼S49, S55∼S58) and prediction set (S12, S24∼S25, S59). PLS-DA was used to perform feature extraction of chemical components by statistical software (Simca 14.0; Umetrics, Andover, MA, United States). The fingerprint peaks with VIP value greater than 1 and closely related to anti-SARS-CoV2 M^pro^ activity were integrated to establish chemical pattern recognition model by PLS-DA. The discriminant classification model was obtained by the training set and validated by the testing set and the prediction set. Furthermore, the classification accuracy, precision, sensitivity and specificity analysis were applied to evaluate the model performance. Values of accuracy close or equal to 100%, precision, sensitivity and specificity of 1.00 show well discriminant ability (Badaró et al., 2020; Oliveira et al., 2021).

### UPLC/Q-TOF-MS Instrument and Conditions

UPLC/Q-TOF-MS analysis was performed on SCIEX Shimadzu RUO LC system coupled with SCIEX X500R QTOF (AB SCIEX, Foster City, CA, United States) with an ESI interface. The separation column and gradient program of mobile phase were the same as the above UHPLC condition. Optimized source and gas parameters were follows: both of the nebulizing gas (Gas 1) and heater gas (Gas 2) were 50 psi, the curtain gas was 35 psi, ion source temperature was 500°C. TOF-MS survey was scanned with Information Dependent Acquisition mode, the ion spray voltage was 5500 V. Q-TOF/MS were set as follows: mass range, 100–1,500; declustering potential, 50 V; collision energy, 10 V. Data was processed by SCIEX OS software.

### Validation of the Quantitative Method

For the calibration curve, analytical standard solutions with a series of concentration levels (*X*, μg·mL^−1^) were prepared to acquire corresponding peak area (*Y*). The calibration curve was constructed by plotting *Y* against *X*, and correlation coefficient was determined by weighted (1/*X* ^2^) least-squares linear regression analysis. The limit of detection (LOD) and limit of quantification (LOQ) were the concentration of analytical standard solutions with a signal-to-noise ratio (S/N) of 3 and 10, respectively. The repeatability was measured by six bunches of samples prepared in parallel, and expressed as the relative standard deviation (RSD). The analysis of intra-day and inter-day precision were measured by assaying the analytical standard solutions within 1 day and three different days, respectively. Testing for stability was carried out in 0, 2, 4, 8, 16, 24, 48, and 72 h. The recovery was measured by adding the standard references to the sample at the same concentration as the known amount in sample (He et al., 2021).

### Data Analysis

All data points represent the mean ± SEM. The concentration-inhibition rate curves were generated using GraphPad software fitted by a non-linear logistic equation (Ver 9.0, GraphPad Software Inc., San Diego, CA, United States). Statistical significance between groups was calculated using one-way ANOVA followed by post-hoc Dunnett’s multiple comparisons. A *P* value below 0.05 was considered to be statistically significant.

## Results

### Optimization of UHPLC Condition

**FIGURE 2:**
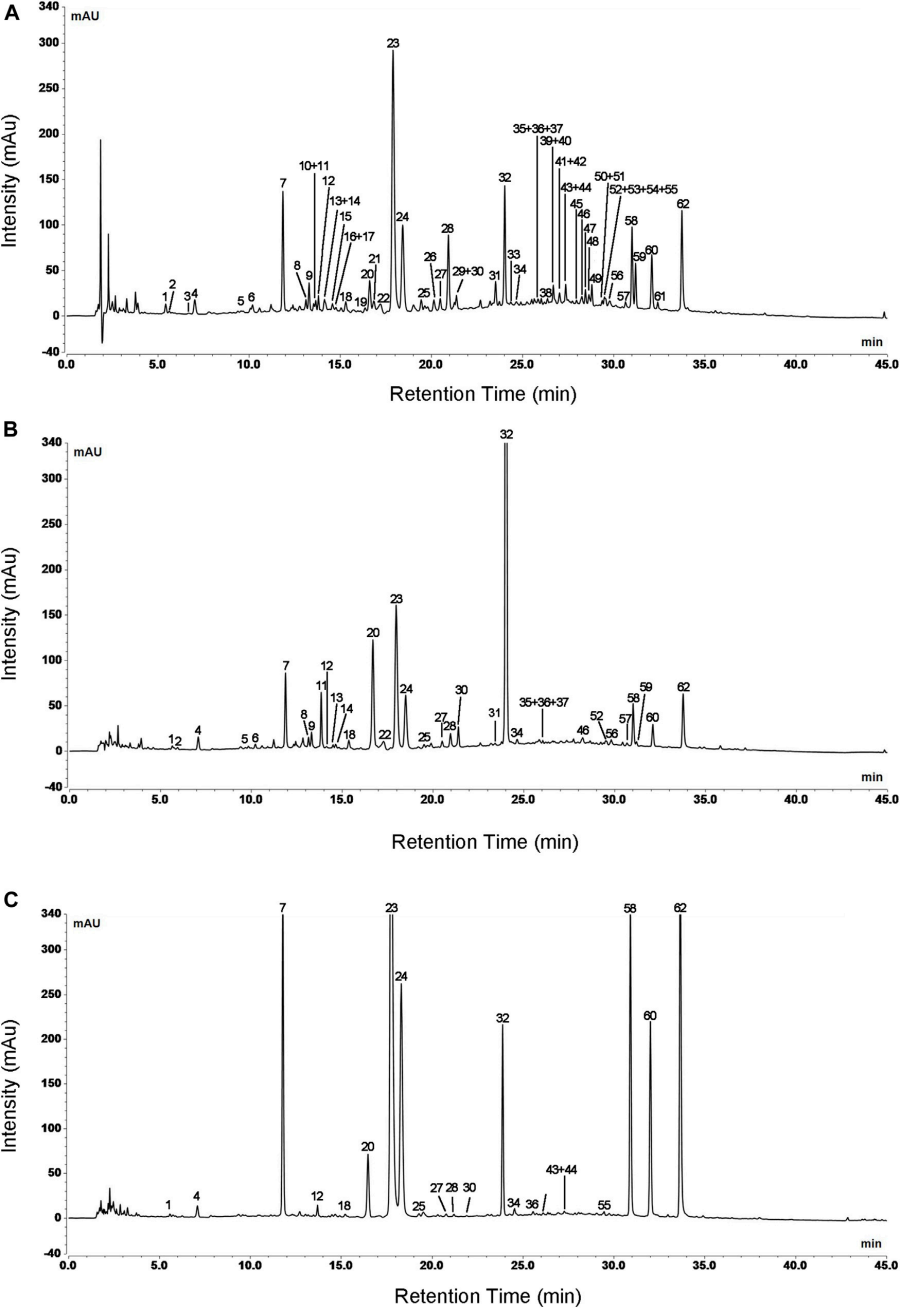
Typical UPLC profiles of cultivated L. japonicae flos **(A)**, wild L. japonicae flos **(B)**, and L. flos **(C)**. Numbers indicated a total of 62 peaks detected.

Based on clinical practice of traditional Chinese medicine that decoction is the most common method of administration, water reflux extraction was applied to extract L. japonicae flos and L. flos samples. UHPLC conditions were optimized to achieve the most useful chemical information and a satisfactory separation in the fingerprint chromatograms of L. japonicae flos and L. flos. Particle size of the column at 2.7 μm possessed best separation effect of chromatographic peaks. Acetonitrile-water mobile phase system showed more powerful resolution than methanol-water system. Furthermore, when the mobile phase was added with formic acid, the shape and symmetry of chromatographic peaks were significantly improved. In addition, the column temperature also affects the chromatographic separation. As a result, 0.1% aqueous formic acid/acetonitrile at the flow rate of 0.9 ml/min, column temperature was maintained at 15°C and a strong UV absorption at 240 nm were chosen for the determination with most peaks on the chromatogram appearing within 45 min. Typical UPLC profiles of cultivated L. japonicae flos, wild L. japonicae flos, and L. flos are shown in Figure 2.

### Validation of the Qualitative Method

Considering the characteristics of fingerprint profiles, the optimum method was validated for precisions, repeatability, and stability. The retention times (RT) and peak areas (PA) of seven common peaks detected were used to evaluate the fingerprint. Analysis of precision was measured by injecting six replicate injections of the same L. japonicae flos sample solutions. Variations were expressed by the RSD of PA and RT of seven common peaks, which were less than 0.1 and 0.98%, respectively (Supplementary Table S2). The repeatability was measured by six bunches of samples prepared in parallel, and the RSDs of PA and RT were less than 0.12 and 2.96% (Supplementary Table S2). Testing for stability was carried out in 0, 2, 4, 8, 16, 24, 48 and 72 h with RSDs of PA and RT no more than 0.56 and 2.43%, respectively (Supplementary Table S2). The results indicated that developed UHPLC method is precise, reliable and stable enough for fingerprint analysis.

### Molecular Feature Extraction Based on UHPLC Profiles

**FIGURE 3:**
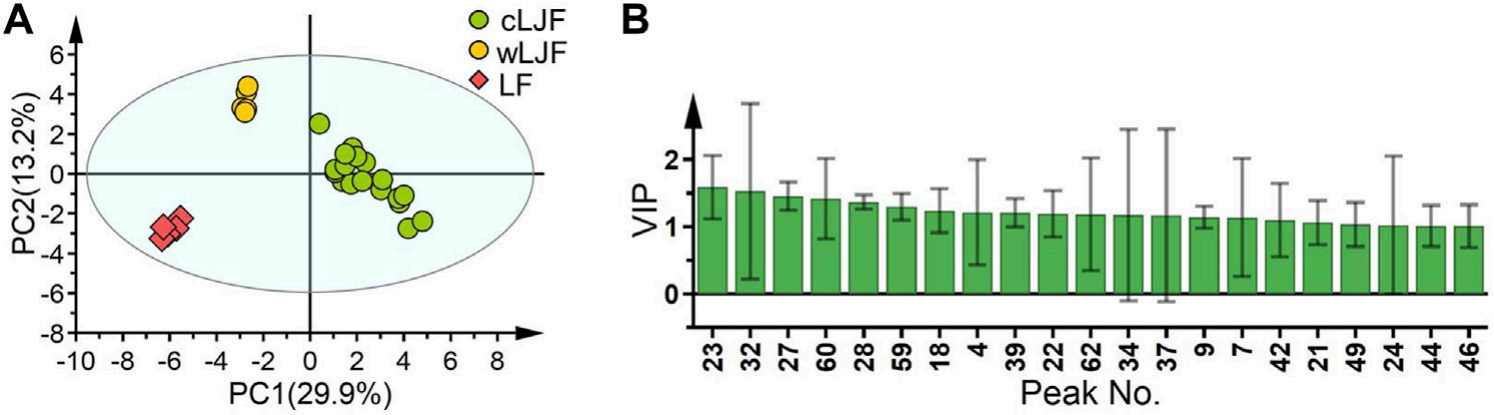
Molecular feature extraction by PLS-DA based on chemical information. **(A)** PLS-DA score plot of training set samples. Cluster 1 (green circles): cultivated L. japonicae flos; Cluster 2 (yellow circles): wild L. japonicae flos; Cluster 3 (red squares): L. flos. **(B)** Chromatographic peaks with VIP value > 1.

The fingerprints of 59 batches of L. japonicae flos and L. flos samples were collected under the optimized UHPLC conditions. 28 batches of training set samples were applied to extract feature peaks by PLS-DA (Figure 3A). The contributions of first two principal components are 29.9 and 13.2%, respectively (Figure 3A). VIP values produced by PLS-DA gave a good reference for identifying peaks that had a significant impact on the classification. In this study, 21 components with VIP values greater than 1 were selected as discriminate markers for authentication of cultivated L. japonicae flos, wild L. japonicae flos and L. flos (Figure 3B). The significant level was follows: peak 23 > 32 > 27 > 60 > 28 > 61 > 18 > 4 > 39 > 20 > 62 > 34 > 37 > 9 > 7 > 42 > 21 > 49 > 24 > 44 > 46.

### L. japonicae Flos and L. Flos Inhibit the SARS-CoV2 M^pro^ Activity

**FIGURE 4:**
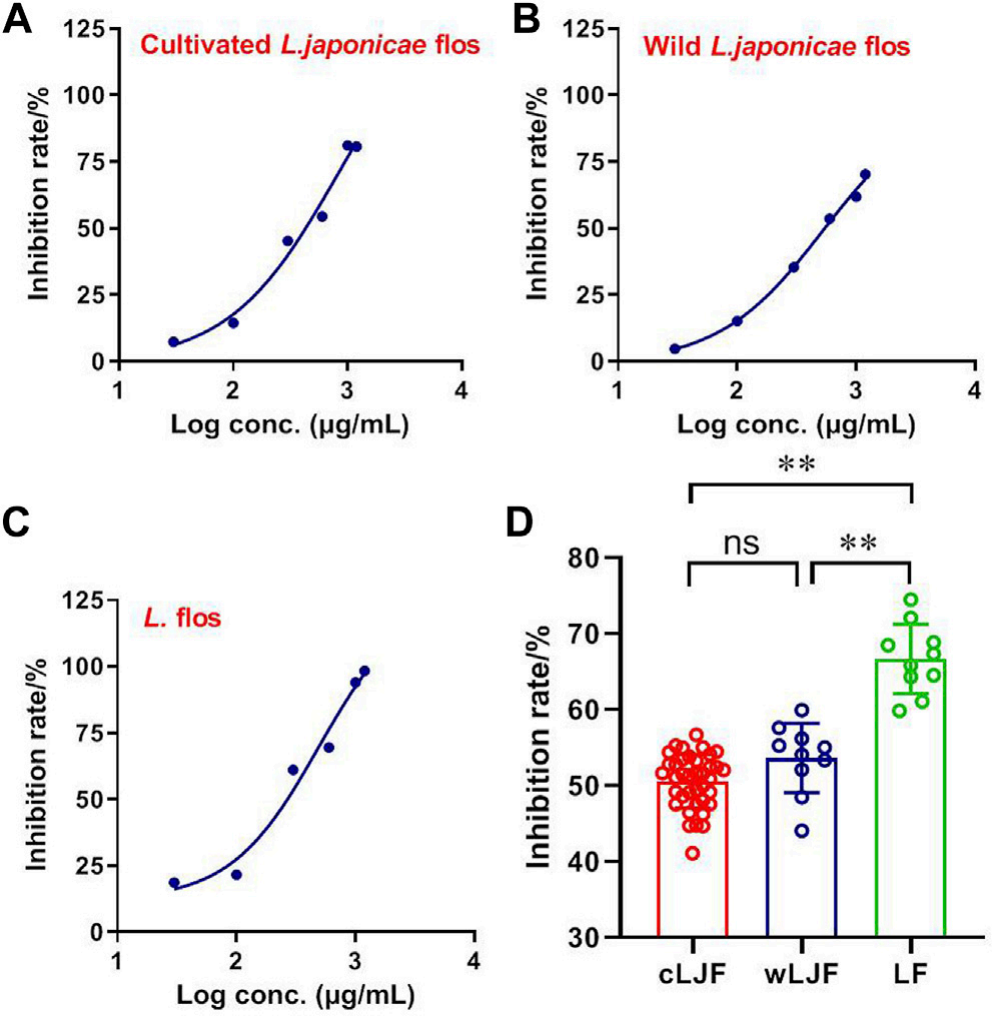
The inhibitory efficacy of cultivated L. japonicae flos, wild L. japonicae flos and L. flos on SARS-CoV2 M^pro^ activity. IC_50_ of cultivated L. japonicae flos **(A)**, wild L. japonicae flos **(B)**, and L. flos **(C)**. **(D)** Inhibition of SARS-CoV2 M^pro^ activity by 59 batches of samples. ^**^ *p* < 0.01, cultivated L. japonicae flos or wild L. japonicae flos group *vs.* L. flos group.

M^pro^ is the viral protease involved in production of functional polyproteins required for SARS-CoV2 replication (Boopathi et al., 2021). The inhibitory activity of L. japonicae flos and L. flos were characterized in dosage gradient by FRET enzymatic assay. Cultivated L. japonicae flos, wild L. japonicae flos and L. flos showed inhibitory activity of SARS-CoV2 M^pro^ in a dose-dependent manner with IC_50_ of 831.30 μg/ ml, 528.90 μg/ml and 495.70 μg/ ml, respectively (Figure 4A-C). Specifically, the inhibition rate of L. flos on SARS-CoV2 M^pro^ activity reached 98.34% (Figure 4C). Then, the inhibition rate of 59 batches of *Lonicera* samples against SARS-CoV2 M^pro^ was measured at the 600 μg/ ml. The results indicated that the inhibition rate of L. flos (66.68 ± 1.44%) was significantly higher compared to cultivated and wild L. japonicae flos (50.58 ± 0.57%, 53.68 ± 1.44%) (Figure 4D). The better inhibitory effect of L. flos on SARS-CoV2 M^pro^ activity implied that the components with higher content in L. flos may contribute to SARS-CoV2 inhibition effect compared to L. japonicae flos.

### Discovery and Characterization of Compounds Related to anti-SARS-CoV2 M^pro^ Activity

The grey relational analysis was used to investigate the spectrum-effect relationships between all chromatographic peaks and inhibitory effect of SARS-CoV2 M^pro^ of 59 samples of *Lonicera*. The relational degree was calculated to sort the potential bioactive components associated with anti-SARS-CoV2 M^pro^ activity, which closer to 1 means the higher efficacy contribution of the corresponding component. A total of 15 main peaks with the relevancy degrees greater than 0.979 were selected. The ranking of relevancy degree was the number of peak 23 > 7 > 24 > 62 > 60 > 18 > 58 > 20 > 4 > 22 > 28 > 61 > 32 > 27 > 9 (Table 1).

**TABLE 1:** Grey correlation analysis and component analysis results.

No.	Peaks	Correlation degree	Formula	Measured mass	Mass error (mDa)	MS fragmentation	Identification
1	23*	0.994	C_16_H_18_O_9_	353.0854	−2.4	191.0551 179.0348 173.0348 161.0246 135.0448	Chlorogenic acid
2	7*	0.993	C_16_H_18_O_9_	353.0882	0.4	191.0555 179.0354 173.0458 135.0449	Neochlorogenic acid
3	24*	0.993	C_16_H_18_O_9_	353.0861	−1.7	191.0559 179.0347 135.0450	Cryptochlorogenic acid
4	62*	0.991	C_25_H_24_O_12_	515.1193	−0.2	353.0924 191.0556 179.0340 173.0464 135.0454	4,5-*O*-dicaffeoyl quinic acid
5	60*	0.99	C_25_H_24_O_12_	515.1194	−0.1	353.0868 191.0563 179.0351 173.0454 135.0446	3,5-*O*-dicaffeoyl quinic acid
6	18*	0.99	C_17_H_26_O_11_	451.1472	1.5	243.0874 141.0568 101.0244	Morroniside
7	58*	0.99	C_25_H_24_O_12_	515.1180	−1.5	353.0880 191.0567 179.0349 173.0457 135.0459	3,4-*O*-dicaffeoyl quinic acid
8	20*	0.986	C_16_H_22_O_10_	373.1143	0.3	167.0720 149.0612 123.0451	Swertiamarin
9	4	0.987	C_16_H_22_O_11_	389.1069	−2.0	165.0549 121.0668	Secologaniside isomer
10	22	0.988	C_16_H_22_O_11_	389.1099	1.0	165.0533 121.0672	Secologaniside
11	28*	0.985	C_16_H_22_O_9_	403.1232	−1.4	357.1199 195.0591 125.0265	Sweroside
12	61*	0.982	C_34_H_46_O_19_	757.2539	−2.2	595.2061 525.1618 493.1698	Centauroside
13	32*	0.981	C_17_H_24_O_11_	403.1227	−1.9	165.0580 121.0318	Secoxyloganin
14	27	0.979	C_17_H_24_O_10_	433.1353	0.2	225.0793 101.0265	Secologanin
15	9	0.979	C_16_H_24_O_10_	375.1276	−2.1	213.0766 169.0867 151.0741	Demethylsecologanol

Note: * identified by comparing with analytical standards.

With the purpose of identifying the fifteen components, UPLC/Q-TOF-MS analysis was applied. Peaks 23, 7, and 24 all showed same [M−H]^−^ ions at *m/z* 353.0854 (C_16_H_18_O_9_), and obvious fragment ions of quinic acids moiety and caffeic acid moiety at *m/z* 191.0551 and 179.0348, respectively. In addition, a dehydrated quinic acid moiety at *m/z* 173.0348, a dehydrated caffeic acid moiety at *m/z* 161.0246 and a decarboxylated caffeic acid moiety at *m/z* 135.0448 were also detected. Accordingly to the reported polarity, retention behavior of the isomers and further validated by reference standards, peaks 23, 7, and 24 were unambitiously identified as 3-*O*-caffeoyl quinic acid (chlorogenic acid), 5-*O*-caffeoyl quinic acid (neochlorogenic acid) and 4-*O*-caffeoyl quinic acid (cryptochlorogenic acid) (Qi et al., 2009; Cai et al., 2020; Zhang et al., 2021). Peaks 62, 58, and 60 yielded an identical [M−H]^−^ ion at *m/z* 515.1193 (C_25_H_24_O_12_). As compared to peaks 23, 7, and 24, peaks 62, 58 and 60 had one more caffeoyl units. They are a group of dicaffeoylquinic acid isomers, shared similar fragments ions as peaks 62, 58, and 60, such as *m/z* 353.0924, 191.0556, 179.0340 and 173.0464. Moreover, by comparison of retention times, mass spectra with reference substances, peaks 62, 58, and 60 could be unambitiously assigned as 4,5-*O*-dicaffeoyl quinic acid, 3,4-*O*-dicaffeoyl quinic acid and 3,5-*O*-dicaffeoyl quinic acid (Qi et al., 2009; Cai et al., 2020; Zhang et al., 2021). Peak 18 generated a [M + HCOOH−H]^−^ ion at *m/z* 451.1472 (C_25_H_24_O_12_), and fragment ions at 243.0874, 141.0568, and 101.0244 produced by neutral elimination of glucose unit (162 Da) and RDA cleavages from [M−H]^−^ ion, which further unambitiously identified as morroniside by comparison with reference standard (Cai et al., 2020). Peak 22 and peak 4 both presented [M−H]^−^ ion at *m/z* 389.1099 (C_16_H_22_O_11_), and similar fragment ions at 165.0533 [M−H−C_6_H_10_O_5_−CO_2_]^−^, 121.0668 [M−H−C_6_H_10_O_5_−CO_2_−CO_2_]^−^. By comparing with the retention time of literature information, secologaniside was considered to be the most appropriate candidate of peak 22. Then peak four was tentatively characterized as secologaniside isomer (Qi et al., 2009; Cai et al., 2020; Zhang et al., 2021). Peak 20 yielded [M−H]^−^ ion at *m/z* 373.1143 (C_16_H_22_O_10_), and a series of product ions at *m/z* 167.0720 by successive losses of glucose and CO_2_, at *m/z* 149.0612 by subsequently dehydration from 167.0720, and at *m/z* 123.0451 by loss of CO from 149.0612. Then, it was characterized as swertiamarin accordingly to reference substance data (Cai et al., 2020; Zhang et al., 2021). Although, peak 28 and 32 both displayed parent ion at *m/z* 403.1232, their secondary fragment ions were totally different. The [M + HCOOH−H]^−^ ion at *m/z* 403.1232 and [M−H]^−^ ion at *m/z* 357.1199 was peak 28. Its’ fragment ions at *m/z* 195.0591 and 125.0265 were produced by losses of glucose and RDA cleavages. As for peak 32, 403.1227 was the deprotonated ion, and fragment ions at *m/z* 223.0633, 165.0580 and 121.0318 were generated by successively losses of C_6_H_10_O_5_, COOCH_3_ and CO_2_. And fragment ion at *m/z* 179.0601 was produced by loss of CO_2_ from ion at *m/z* 223.0633. Further, combined with the data from reference substances, peak 28 and 32 were identified as sweroside and secoxyloganin, respectively (Qi et al., 2009; Cai et al., 2020; Zhang et al., 2021). Peak 27 presented [M + HCOOH−H]^−^ ion at *m/z* 433.1353. Neutral loss of a glucose moiety produced the ion at *m/z* 225.0793. Ion at *m/z* 101.0265 was produced by RDA cleavages, accordingly, peak 27 was tentatively characterized as secologanin (Zhang et al., 2021). Peak 61 generated a [M−H]^−^ ion at *m/z* 757.2539 (C_34_H_46_O_19_), and fragment ions at 595.2061, 525.1618, 493.1698 generated by neutral elimination of glucose unit (162 Da), RDA cleavages from [M−H]^−^ ion and successively loss of CH_3_O from ion at *m/z* 525.1618, which further unambitiously identified as centauroside by comparison with reference standard (Pan et al., 2020; Zhang et al., 2021). Peak nine presented [M−H]^−^ ion at *m/z* 375.1276 (C_16_H_24_O_10_), and fragment ions at 213.0766, 169.0867 and 151.0741 produced by losses of glucose unit, HCOOH and H_2_O. By comparing with the retention time and polarity of reported data, peak nine was assigned as demethylsecologanol (Zhang et al., 2021).

### Classification of L. japonicae Flos and L. Flos Samples Using PLS-DA

**FIGURE 5:**
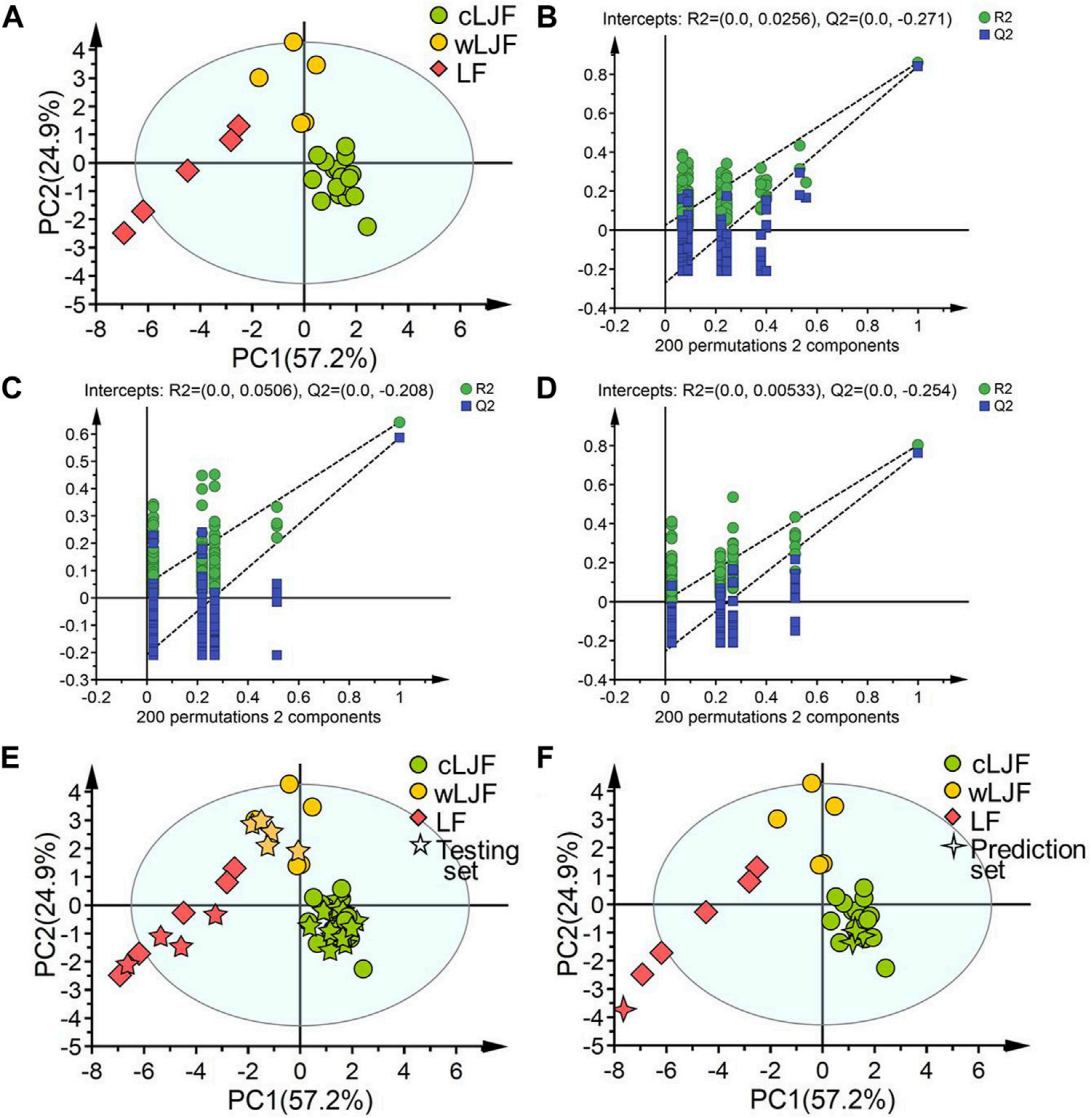
The establishment, validation and prediction of chemical pattern recognition model based on 11 characteristic components. **(A**) PLS-DA score plot of training set samples. **(B)** Permutation tests result of cultivated L. japonicae flos samples. **(C)** Permutation tests result of wild L. japonicae flos samples. **(D)** Permutation tests result of L. flos samples. **(E)** PLS-DA score plot of training set and testing set samples. **(F)** PLS-DA score plot of training set and prediction set samples.

To explore the representative components, 15 potential bioactive components associated with anti-SARS-CoV2 M^pro^ activity were then overlapped with 21 extracted components that played critical role in differentiating cultivated L. japonicae flos, wild L. japonicae flos and L. flos samples, and then 14 components obtained. Combined with the rule that analytical standard is commercially available, 11 bioactive components (peak 23, 7, 24, 62, 60, 18, 58, 20, 28, 61, 32) were chosen to establish a quality evaluation model for *Lonicera* samples using PLS-DA. Training set samples from cultivated L. japonicae flos, wild L. japonicae flos and L. flos were successfully segregated (Figure 5A). Two principal components are obtained by fitting with contributions of 57.2 and 24.9%, respectively (Figure 5A). The established model had excellent goodness of fit (R^2^X = 0.821, R^2^Y = 0.770) and predictive ability (Q^2^ = 0.736). Permutation tests results confirmed that the established model avoided an over-fitting problem for the values of substrate Q^2^ intercept from *R* ^2^ intercept were lower than 0.3 (Figures 5B–D). Next, testing set samples were applied to validate the model. Validation results indicated 27 samples were correctly divided into their own categories with 100% accuracy, which proved the great performance of established model (Figure 5E; Table 2). In order to verify the predictive ability of PLS-DA model, prediction set samples were selected as investigation. As shown in Figure 5F; Table 2, three batches of samples were identified as cultivated cultivated L. japonicae flos and 1 batch of sample was classified as L. flos at the accuracy of 100%. With regards to model performance, the equal to 1.00 value of precision, sensitivity and specificity demonstrated the good capacity of the model to discriminate samples (Table 2). In sum, these results showed that bioactive components based discriminatory strategy was reliable which could be used for quality evaluation as well as quality prediction of cultivated L. japonicae flos, wild L. japonicae flos and L. flos samples.

**TABLE 2:** Classification results and model performance of the PLS-DA model.

Set	Actual categories	Prediction results			Accuracy (%)	Precision	Sensitivity	Specificity
		cLJF	wLJF	LF				
Training set	cLJF	18	0	0	100	1.00	1.00	1.00
	wLJF	0	5	0				
	LF	0	0	5				
Testing set	cLJF	18	0	0	100	1.00	1.00	1.00
	wLJF	0	5	0				
	LF	0	0	4				
Prediction set	cLJF	3	0	0	100	1.00	1.00	1.00
	wLJF	0	0	0				
	LF	0	0	1				

### Quantitative Analysis of Characteristic Components of all Samples

**FIGURE 6:**
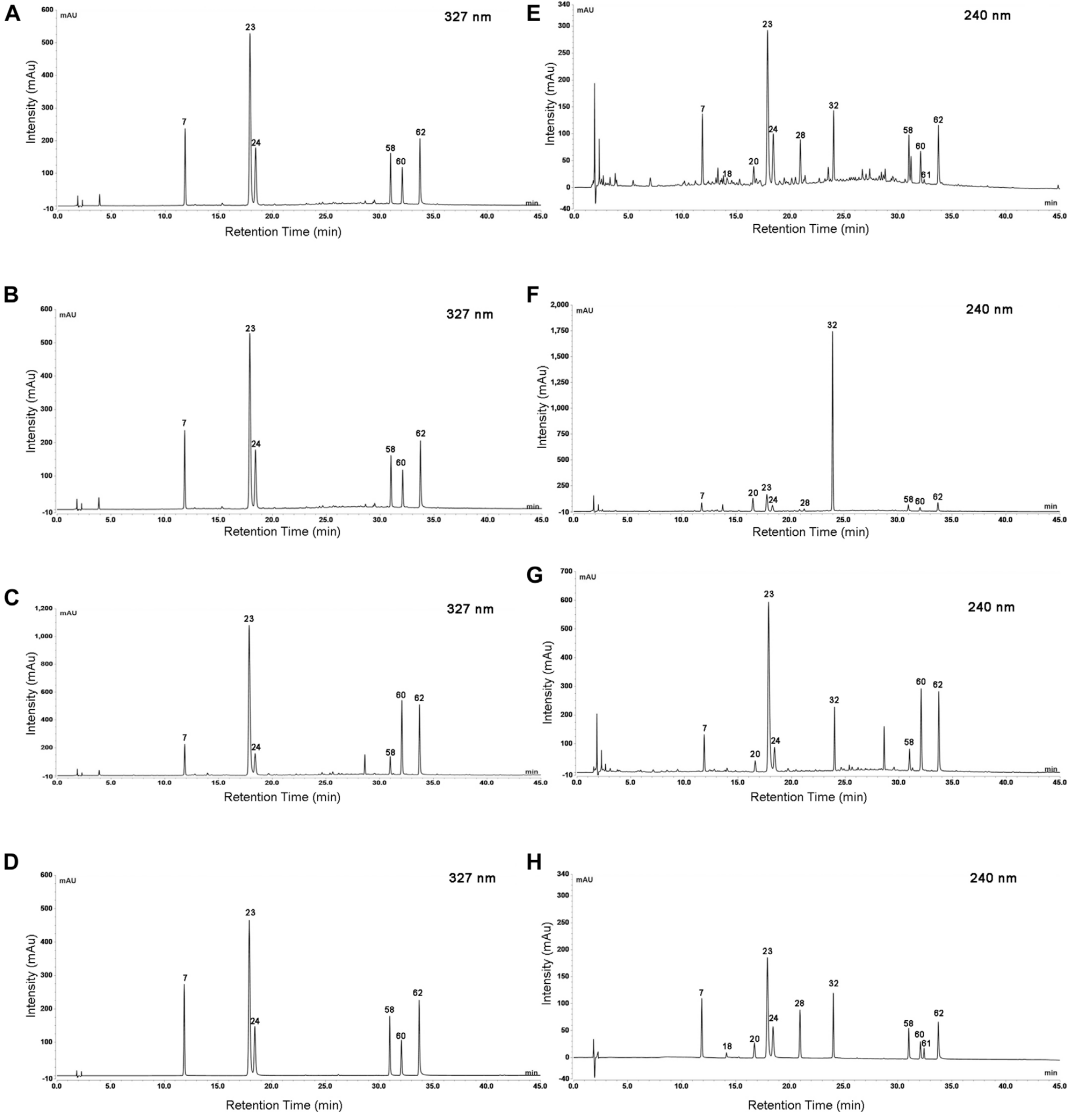
The UHPLC chromatogram of Lonicerae japonicae flos, Lonicerae flos and mixed analytical standards at wavelength of 327 and 240 nm. Cultivated Lonicerae japonicae flos at 327 nm **(A)** and 240 nm **(E)**; Wild Lonicerae japonicae flos at 327 nm **(B)** and 240 nm **(F**); Lonicerae flos at 327 nm **(C**) and 240 nm **(G)**; Mixed analytical standards at 327 nm **(D**) and 240 nm **(H)**. 7. neochlorogenic acid; 18. morroniside; 20. swertiamarin; 23. chlorogenic acid; 24. cryptochlorogenic acid; 28. sweroside; 32. Secoxyloganin; 58. 3,4-*O*-dicaffeoyl quinic acid; 60. 3,5-*O*-dicaffeoyl quinic acid; 61. centauroside; 62. 4,5-*O*-dicaffeoyl quinic acid.

**FIGURE 7:**
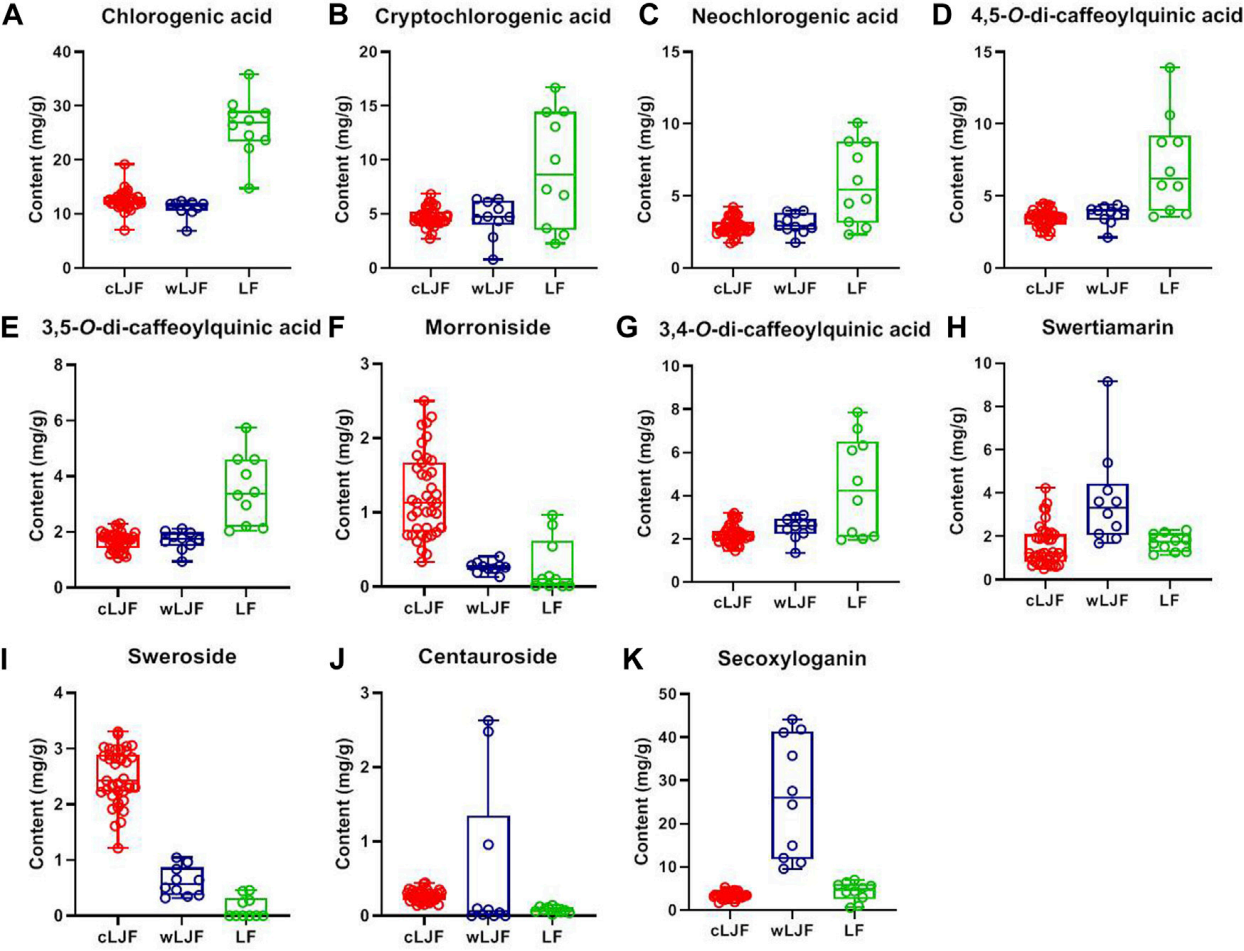
The contents of 11 characteristic components in cultivated L. japonicae flos, wild L. japonicae flos and L. flos. **(A)** Chlorogenic acid. **(B)** Cryptochlorogenic acid. **(C)** Neochlorogenic acid. **(D)** 4,5-*O*-dicaffeoyl quinic acid. **(E)** 3,5-*O*-dicaffeoyl quinic acid. **(F)** Morroniside. **(G)** 3,4-*O*-dicaffeoyl quinic acid. **(H)** Swertiamarin. **(I)** Sweroside. **(J)** Centauroside. **(K)** Secoxyloganin.

Quantitative analysis of characteristic components could reveal the variation of samples and provide reference for the quality standard setting. Considering that eleven characteristic components belong to caffeoylquinic acids (Peak 23, 7, 24, 62, 60, and 58) and iridoid glycosides (Peak 18, 22, 28, 61, and 32), the detection wavelength was set at 327 and 240 nm, respectively (Figure 6). The contents of eleven characteristic components in 59 batches of L. japonicae flos and L. flos samples were summarized in Table 3. Chlorogenic acid was the predominant component in cultivated L. japonicae flos and L. flos samples with the content of 1.21% ± 0.03 and 2.62% ± 0.18, respectively (Table 3). In wild L. japonicae flos samples, secoxyloganin had the highest rate of 2.45% ± 0.43 (Table 3). The contents of caffeoylquinic acids, including chlorogenic acid, cryptochlorogenic acid, neochlorogenic acid, 4,5-*O*-dicaffeoyl quinic acid, 3,5-*O*-dicaffeoyl quinic acid and 3,4-*O*-dicaffeoyl quinic acid, in L. flos samples is about twice of which in L. japonicae flos samples (Table 3; Figures 7A–E,G). While the contents of morroniside and sweroside in cultivated L. japonicae flos was significantly higher than that in wild L. japonicae flos and L. flos samples (Table 3; Figures 7F,I). It is worth mentioning that the contents of morroniside and sweroside in the majority of L. flos samples are close to zero, and they can be recognized as quality marker for L. japonicae flos to distinguish cultivated L. japonicae flos from L. flos and wild L. japonicae flos samples. The content of swertiamarin in wild L. japonicae flos was slightly higher than the other two, but the content of secoxyloganin was significantly higher than cultivated L. japonicae flos and L. flos (Table 3; Figure 7K). As for centauroside, although the average content in wild L. japonicae flos is the highest, it has not been detected in the majority of wild L. japonicae flos and L. flos samples (Table 3; Figure 7J).

**TABLE 3:** The contents of 11 characteristic components in 59 batches of L. japonicae flos and L. flos samples.

No.	P23 (%)	P24 (%)	P7 (%)	P62 (%)	P60 (%)	P18 (%)	P58 (%)	P20 (%)	P28 (%)	P61 (%)	P32 (%)
S1	1.199	0.451	0.277	0.245	0.111	0.078	0.164	0.109	0.224	0.025	0.271
S2	1.246	0.563	0.366	0.437	0.230	0.229	0.309	0.114	0.276	0.031	0.428
S3	1.360	0.613	0.392	0.365	0.184	0.194	0.255	0.050	0.246	0.017	0.325
S4	1.250	0.435	0.26	0.298	0.143	0.112	0.184	0.208	0.242	0.015	0.332
S5	1.263	0.422	0.253	0.357	0.177	0.069	0.215	0.217	0.283	0.022	0.338
S6	1.194	0.510	0.324	0.287	0.131	0.151	0.197	0.064	0.224	0.028	0.353
S7	1.213	0.578	0.38	0.448	0.225	0.202	0.319	0.093	0.326	0.026	0.462
S8	1.443	0.682	0.422	0.435	0.202	0.160	0.298	0.073	0.331	0.021	0.527
S9	1.295	0.45	0.271	0.378	0.190	0.070	0.229	0.194	0.299	0.030	0.335
S10	1.260	0.425	0.253	0.354	0.173	0.080	0.212	0.186	0.304	0.031	0.312
S11	1.236	0.483	0.305	0.359	0.182	0.149	0.238	0.106	0.227	0.034	0.341
S12	1.102	0.523	0.343	0.403	0.201	0.250	0.293	0.092	0.289	0.030	0.420
S13	1.321	0.442	0.264	0.379	0.191	0.095	0.222	0.222	0.306	0.030	0.366
S14	1.199	0.454	0.291	0.249	0.118	0.122	0.168	0.066	0.207	0.020	0.303
S15	1.232	0.471	0.293	0.353	0.180	0.113	0.234	0.127	0.235	0.035	0.343
S16	1.174	0.433	0.267	0.269	0.137	0.117	0.177	0.061	0.192	0.014	0.314
S17	1.370	0.449	0.269	0.377	0.182	0.154	0.224	0.194	0.299	0.033	0.403
S18	1.139	0.417	0.249	0.327	0.156	0.101	0.204	0.185	0.272	0.028	0.318
S19	1.173	0.430	0.258	0.325	0.160	0.115	0.203	0.123	0.230	0.043	0.330
S20	1.072	0.432	0.271	0.327	0.171	0.124	0.219	0.118	0.23	0.025	0.362
S21	1.175	0.491	0.311	0.374	0.188	0.218	0.251	0.076	0.238	0.034	0.378
S22	1.229	0.424	0.25	0.351	0.167	0.099	0.211	0.189	0.296	0.027	0.347
S23	1.321	0.432	0.258	0.370	0.180	0.101	0.217	0.210	0.283	0.031	0.320
S24	1.124	0.465	0.296	0.347	0.176	0.177	0.234	0.122	0.233	0.025	0.382
S25	1.266	0.517	0.322	0.337	0.154	0.167	0.228	0.065	0.222	0.026	0.366
S26	1.133	0.380	0.228	0.309	0.148	0.079	0.188	0.226	0.281	0.036	0.332
S27	1.38	0.415	0.238	0.369	0.182	0.073	0.211	0.353	0.202	0.023	0.276
S28	1.289	0.487	0.297	0.337	0.167	0.170	0.219	0.106	0.216	0.035	0.323
S29	1.022	0.314	0.185	0.285	0.137	0.067	0.167	0.245	0.188	0.024	0.235
S30	1.147	0.481	0.306	0.379	0.192	0.133	0.256	0.116	0.316	0.045	0.408
S31	1.214	0.401	0.243	0.327	0.162	0.086	0.201	0.158	0.250	0.026	0.264
S32	1.271	0.422	0.254	0.291	0.129	0.070	0.186	0.288	0.280	0.017	0.256
S33	1.154	0.363	0.207	0.283	0.125	0.044	0.166	0.331	0.161	0.027	0.164
S34	1.917	0.419	0.224	0.301	0.137	0.050	0.160	0.307	0.178	0.026	0.127
S35	1.171	0.382	0.227	0.309	0.150	0.067	0.184	0.290	0.225	0.026	0.242
S36	1.177	0.378	0.226	0.308	0.152	0.033	0.179	0.301	0.236	0.022	0.258
S37	0.694	0.270	0.172	0.252	0.121	0.049	0.163	0.327	0.168	0.021	0.338
S38	1.180	0.362	0.215	0.303	0.145	0.069	0.180	0.365	0.258	0.012	0.270
S39	1.268	0.387	0.231	0.334	0.161	0.056	0.190	0.331	0.246	0.023	0.268
S40	0.681	0.284	0.175	0.212	0.094	0.013	0.134	0.413	0.037	0.096	4.18
S41	1.210	0.639	0.393	0.425	0.203	0.029	0.302	0.305	0.064	0.248	4.415
S42	1.060	0.453	0.278	0.34	0.166	0.024	0.225	0.212	0.105	0.008	0.957
S43	1.137	0.472	0.293	0.367	0.183	0.027	0.244	0.247	0.096	0.010	1.205
S44	1.183	0.077	0.251	0.098	0.137	0.026	0.263	0.061	0.051	-	1.101
S45	1.237	0.637	0.397	0.438	0.213	0.024	0.311	0.19	0.046	0.263	4.112
S46	1.186	0.547	0.331	0.390	0.173	0.041	0.266	0.539	0.032	−	3.575
S47	1.110	0.476	0.286	0.400	0.177	0.032	0.264	0.914	0.035	−	2.755
S48	1.196	0.612	0.378	0.407	0.198	0.028	0.290	0.168	0.065	−	2.444
S49	1.037	0.434	0.27	0.317	0.153	0.019	0.259	0.361	0.084	−	1.495
S50	2.367	0.307	0.232	0.355	0.221	0.083	0.195	0.31	−	−	0.419
S51	2.867	0.367	0.278	0.401	0.332	0.096	0.2	0.115	−	0.010	0.512
S52	2.734	1.45	0.874	0.876	0.462	−	0.609	0.184	−	−	0.583
S53	3.022	1.308	0.766	1.062	0.574	−	0.71	0.23	0.024	−	0.058
S54	3.582	0.675	0.479	0.575	0.407	0.054	0.23	0.126	−	0.011	0.561
S55	2.854	1.668	1.007	0.873	0.461	−	0.632	0.166	−	0.014	0.696
S56	2.458	1.443	0.88	0.669	0.344	−	0.469	0.128		0.012	0.640
S57	2.218	1.005	0.61	0.569	0.297	0.010	0.378	0.22	0.044	−	0.433
S58	1.474	0.226	0.447	1.391	0.203	0.011	0.786	0.212	0.028	−	0.295
S59	2.643	0.767	0.427	0.374	0.213	0.014	1.614	0.152	0.046	−	0.095

Note: under the limit of quantitation; P23. chlorogenic acid; P24. cryptochlorogenic acid; P7. neochlorogenic acid; P62. 4,5-*O*-dicaffeoyl quinic acid; P60. 3,5-*O*-dicaffeoyl quinic acid; P18. morroniside; P58. 3,4-*O*-dicaffeoyl quinic acid; P20. swertiamarin; P28. sweroside; P61. centauroside; P32. secoxyloganin.

### Validation of the Quantitative Method

Under the optimum UHPLC conditions, the calibration curves of eleven characteristic components showed good linearity with correlation coefficient values more than 0.9999 (Supplementary Table S3). The LOD (S/N 3) and LOQ (S/N 10) for eleven components were ranged from 0.46 to 4.44 μg/ ml and 1.25–11.79 μg/ ml, respectively (Supplementary Table S3). The repeatability was assessed by preparing six bunches of samples in parallel, and the RSD for the eleven components were less than 2.89% (Supplementary Table S3). The RSD values of the intra-day and inter-days precisions were in the range of 0.15–0.83% and 0.05–0.62%, respectively (Supplementary Table S4). Sample solution showed good stability for almost 72 h with the RSD values less than 0.37% (Supplementary Table S4). The recovery values of eleven compounds were in the range of 95.52–104.23% (Supplementary Table S4). These results demonstrated that the established method was accurate and reliable for the determination of eleven characteristic components in *Lonicera* samples.

## Discussion

L. japonicae flos is an extremely used heat-clearing and detoxifying botanical drug, which has displayed significant effect on the treatments of SARS, influenza A and COVID-19 in 2003, 2009 and 2020 (Yang et al., 2017; Xiao et al., 2020). In the present study, the detoxifying efficacy of L. japonicae flos was demonstrated by anti-SARS-CoV2 M^pro^ activity. SARS-CoV2 is the etiological agent responsible for the global COVID-19 outbreak (Zhu et al., 2020). After SARS-CoV2 invades the cell, it will immediately utilize the substance in the cell to synthesize two polyproteins, and encode M^pro^. The M^pro^ plays a pivotal role in the proteolytic process that releases the functional polypeptides of Spike, Membrane, Envelop, Nucleoprotein, replicase and polymerase from polyproteins (Boopathi et al., 2021; Mittal et al., 2021). Accordingly, 2019-nCoV M^pro^ is the critical protease for SARS-CoV2 replication. The anti-SARS-CoV2 activity could be revealed by inhibitory effect on M^pro^ (Dai et al., 2020).

The safety and efficacy of botanical drug are key components of their quality. Accordingly, the contents of components closely related to the safety and efficacy of medicines become the important basis for quality evaluation (Wu et al., 2018). However, the relationship between efficacy and specific components in medicines remain to be systematically illustrated. In this study, we focused on the anti-SARS-CoV2 activity of L. japonicae flos to explore its characteristic components which related to detoxifying efficacy and reflected the chemical basis of L. japonicae flos in whole. Eleven characteristic components were screened, including six caffeoylquinic acids (chlorogenic acid, neochlorogenic acid, cryptochlorogenic acid, 4,5-*O*-dicaffeoyl quinic acid, 3,5-*O*-dicaffeoyl quinic acid and 3,4-*O*-dicaffeoyl quinic acid) and five iridoid glycosides (morroniside, swertiamarin, sweroside, centauroside, secoxyloganin). From the perspective of correlation degree with efficacy, six caffeoylquinic acids may have more significant effect on inhibiting M^pro^ activity compared to five iridoid glycosides. Interestingly, caffeoylquinic acid isomers could have better anti-SARS-CoV2 M^pro^ activity than dicaffeoylquinic acid isomers for their higher correlation degree. These results are consistent with the *in silico* studies that chlorogenic acid showed the excellence scores and interactions inside the binding site of SARS-CoV2 M^pro^ (El Gizawy et al., 2021). 3,5-*O*-dicaffeoyl quinic acid was making superior affinity towards M^pro^ as well, which is better than standard drug Remdesivir (Shah et al., 2021). As for discrimination between L. japonicae flos and L. flos, the VIP values of five iridoid glycosides are relatively higher. Compared to L. flos, the contents of five iridoid glycosides are relatively higher in L. japonicae flos samples, which are in accordance with the published results (Chen et al., 2007). Furthermore, it is reported that iridoid glycoside component could be used as a marker to distinguish L. japonicae flos and L. flos for the amount difference (Zhang et al., 2019). However, more pharmacological data about iridoid glycosides are also needed for completely quality evaluation of L. japonicae flos.

As the commonly used folk medicine, L. japonicae flos is cultivated or naturally distributed in most provinces of China (Li et al., 2019). But little attention has been paid to the comparison of quality difference between wild and cultivated L. japonicae flos. Our results indicated that the contents of six caffeoylquinic acids in cultivated and wild L. japonicae flos are almost the same. While the contents of five iridoid glycosides various, which may account for that cultivated and wild L. japonicae flos clustered in two regions in chemical pattern recognition model (Figure 4). However, there was no significant difference between their anti-SARS-CoV2 M^pro^ activities. Therefore, growth mode had little effect on the quality of L. japonicae flos.

## Conclusion

In summary, we demonstrate that both of L. japonicae flos and L. flos possess anti-SARS-CoV2 effect from the perspective of inhibiting M^pro^ activity. We further screened eleven characteristic components which are closely related to anti-SARS-CoV2 activity and display critical role in classifying L. japonicae flos and L. flos. Based on the characteristic components, chemical pattern recognition model of classifying L. japonicae flos and L. flos was successfully established. The proposed strategy provided an efficient tool for quality analysis of traditional Chinese medicine.

## Data Availability

The original contributions presented in the study are included in the article/Supplementary Material, further inquiries can be directed to the corresponding authors.

## Abbreviations

COVID-19, corona virus disease 2019; FRET, fluorescence resonance energy transfer; IC_50_, half-maximal inhibitory concentration; L. flos, Lonicerae flos (*Lonicera macrantha* (D.Don) Spreng.); L. japonicae flos, Lonicerae japonicae flos (*Lonicera japonica* Thunb.); LOD, limit of detection; LOQ, limit of quantification; Mpro, main protease; PA, peak areas; PLS-DA, partial least squares-discriminant analysis; RSD, relative standard deviation; RT, retention times; SARS-CoV2, severe acute respiratory syndrome coronavirus 2; S/N, signal-to-noise ratio; UHPLC, ultra-high performance liquid chromatography; UPLC/Q-TOF-MS, ultra-performance liquid chromatography coupled with quadrupole time-of-flight mass spectrometry; VIP, variable importance in projection.
