# Supplementary material for: Chemical Pattern Recognition for Quality Analysis of Lonicerae Japonicae Flos and Lonicerae Flos Based on Ultra-High Performance Liquid Chromatography and Anti-SARS-CoV2 Main Protease Activity
Source: Front Pharmacol. 2022 Jan 4;12:810748. doi: 10.3389/fphar.2021.810748 (PMC8764198; doi:10.3389/fphar.2021.810748)
Supplement: Supplementary file 1 [file DataSheet1.docx]

**Chemical pattern recognition for quality analysis of Lonicerae Japonicae Flos and Lonicerae Flos based on ultra-high performance liquid chromatography and anti-SARS-CoV2 main protease activity**

Lifei Gu^1,2,3†^ , Xueqing Xie ^3,†^, Bing Wang^1,2^, Yibao Jin^1,2^, Lijun Wang^1,2^, Guo Yin^1,2^, Jue Wang^1,2^, Kaishun Bi^3,*^, and Tiejie Wang ^1,2,3*^

*^1^ NMPA Key Laboratory for Quality Research and Evaluation of Traditional Chinese Medicine, Shenzhen Institute for Drug Control, Shenzhen, Guangdong, 518057, China*

*^2^ Shenzhen Key Laboratory of Drug Quality Standard Research, Shenzhen Institute for Drug Control, Shenzhen, Guangdong, 518057, China*

*^3^ School of Pharmacy, Shenyang Pharmaceutical University, Shenyang, Liaoning, 110016, China*

**Supplementary Tables**

**Table S1** List of the L. japonicae flos and L. flos samples analyzed in this study.

| No. | Species | Collecting location | Growth status |
| --- | --- | --- | --- |
| S1 | L. japonicae flos | Henan Province | Cultivated |
| S2 | L. japonicae flos | Henan Province | Cultivated |
| S3 | L. japonicae flos | Henan Province | Cultivated |
| S4 | L. japonicae flos | Henan Province | Cultivated |
| S5 | L. japonicae flos | Henan Province | Cultivated |
| S6 | L. japonicae flos | Henan Province | Cultivated |
| S7 | L. japonicae flos | Henan Province | Cultivated |
| S8 | L. japonicae flos | Henan Province | Cultivated |
| S9 | L. japonicae flos | Henan Province | Cultivated |
| S10 | L. japonicae flos | Henan Province | Cultivated |
| S11 | L. japonicae flos | Henan Province | Cultivated |
| S12 | L. japonicae flos | Henan Province | Cultivated |
| S13 | L. japonicae flos | Henan Province | Cultivated |
| S14 | L. japonicae flos | Hebei Province | Cultivated |
| S15 | L. japonicae flos | Hebei Province | Cultivated |
| S16 | L. japonicae flos | Hebei Province | Cultivated |
| S17 | L. japonicae flos | Hebei Province | Cultivated |
| S18 | L. japonicae flos | Hebei Province | Cultivated |
| S19 | L. japonicae flos | Hebei Province | Cultivated |
| S20 | L. japonicae flos | Hebei Province | Cultivated |
| S21 | L. japonicae flos | Hebei Province | Cultivated |
| S22 | L. japonicae flos | Hebei Province | Cultivated |
| S23 | L. japonicae flos | Hebei Province | Cultivated |
| S24 | L. japonicae flos | Hebei Province | Cultivated |
| S25 | L. japonicae flos | Hebei Province | Cultivated |
| S26 | L. japonicae flos | Hebei Province | Cultivated |
| S27 | L. japonicae flos | Shandong Province | Cultivated |
| S28 | L. japonicae flos | Shandong Province | Cultivated |
| S29 | L. japonicae flos | Shandong Province | Cultivated |
| S30 | L. japonicae flos | Shandong Province | Cultivated |

(continued on next page)

**Table S1** (continued)

| No. | Species | Collecting location | Growth status |
| --- | --- | --- | --- |
| S31 | L. japonicae flos | Shandong Province | Cultivated |
| S32 | L. japonicae flos | Shandong Province | Cultivated |
| S33 | L. japonicae flos | Shandong Province | Cultivated |
| S34 | L. japonicae flos | Shandong Province | Cultivated |
| S35 | L. japonicae flos | Shandong Province | Cultivated |
| S36 | L. japonicae flos | Shandong Province | Cultivated |
| S37 | L. japonicae flos | Shandong Province | Cultivated |
| S38 | L. japonicae flos | Shandong Province | Cultivated |
| S39 | L. japonicae flos | Shandong Province | Cultivated |
| S40 | L. japonicae flos | Hubei Province | Wild |
| S41 | L. japonicae flos | Hubei Province | Wild |
| S42 | L. japonicae flos | Hubei Province | Wild |
| S43 | L. japonicae flos | Hubei Province | Wild |
| S44 | L. japonicae flos | Hubei Province | Wild |
| S45 | L. japonicae flos | Hubei Province | Wild |
| S46 | L. japonicae flos | Hubei Province | Wild |
| S47 | L. japonicae flos | Hubei Province | Wild |
| S48 | L. japonicae flos | Hubei Province | Wild |
| S49 | L. japonicae flos | Hubei Province | Wild |
| S50 | L. flos | HunanProvince | Wild |
| S51 | L. flos | HunanProvince | Wild |
| S52 | L. flos | HunanProvince | Wild |
| S53 | L. flos | HunanProvince | Wild |
| S54 | L. flos | Guangdong Province | Wild |
| S55 | L. flos | Anhui Province | Wild |
| S56 | L. flos | Anhui Province | Wild |
| S57 | L. flos | Anhui Province | Wild |
| S58 | L. flos | Guizhou Province | Wild |
| S59 | L. flos | Sichuan Province | Wild |

**Table S2** Precision, repeatability and stability of seven common peaks of 59 batches of L. japonicae flos and L. flos samples

| Common peaks | Precision  (RSD%, *n* = 6) | | Repeatability  (RSD%, *n* = 6) | | Stability  (RSD%, *n* = 8) | |
| --- | --- | --- | --- | --- | --- | --- |
|  | Peak area | Retention time | Peak area | Retention time | Peak area | Retention time |
| 7 | 0.10 | 0.30 | 0.09 | 2.01 | 0.36 | 0.36 |
| 20 | 0.05 | 0.98 | 0.12 | 1.41 | 0.56 | 2.43 |
| 23 | 0.07 | 0.19 | 0.09 | 2.53 | 0.52 | 0.24 |
| 24 | 0.09 | 0.34 | 0.09 | 2.17 | 0.52 | 0.41 |
| 32 | 0.01 | 0.26 | 0.06 | 1.20 | 0.25 | 0.68 |
| 58 | 0.02 | 0.67 | 0.04 | 2.44 | 0.26 | 1.67 |
| 62 | 0.02 | 0.80 | 0.04 | 2.96 | 0.28 | 0.82 |

**Table S3** The calibration curves, LODs, LOQs and repeatability of the 11 characteristic components

| Components | Regressive equation | R2 | LOD(μg/mL) | LOQ(μg/mL) | Repeatability RSD (%) |
| --- | --- | --- | --- | --- | --- |
| Chlorogenic acid | *Y*=264.09*X*+1.7803 | 0.9997 | 0.88 | 2.63 | 2.71 |
| Cryptochlorogenic acid | *Y*=232.01*X*+0.4936 | 0.9997 | 0.94 | 2.35 | 1.36 |
| Neochlorogenic acid | *Y*=278.31*X*+0.3739 | 0.9999 | 0.46 | 1.25 | 1.01 |
| 4,5-*O*-dicaffeoyl quinic acid | *Y*=353.69*X*-6.5854 | 0.9992 | 0.57 | 1.48 | 1.68 |
| 3,5-*O*-dicaffeoyl quinic acid | *Y*=290.74*X*-1.5497 | 0.9993 | 0.69 | 2.13 | 1.31 |
| Morroniside | *Y*=58.852*X*+0.0157 | 0.9994 | 4.44 | 11.79 | 2.73 |
| 3,4-*O*-dicaffeoyl quinic acid | *Y*=348.04*X*-3.3421 | 0.9996 | 0.51 | 1.71 | 0.99 |
| Swertiamarin | *Y*=147.78*X*+0.0616 | 0.9995 | 2.34 | 7.81 | 2.34 |
| Sweroside | *Y*=143.39*X*+0.1370 | 0.9995 | 2.11 | 6.84 | 2.89 |
| Centauroside | *Y*=109.19*X*+0.0418 | 0.9994 | 1.94 | 6.28 | 2.64 |
| Secoxyloganin | *Y*=132.98*X*+0.1415 | 0.9995 | 1.68 | 5.59 | 1.31 |

**Table S4** The intra-day, inter-day precision, stability and recovery of the 11 characteristic components

| Components | Intra-day Precision (RSD%) | Inter-day Precision (RSD%) | Stability (RSD%) | Average recovery (%) | Recovery (RSD%) |
| --- | --- | --- | --- | --- | --- |
| Chlorogenic acid | 0.16 | 0.28 | 0.14 | 98.72 | 0.64 |
| Cryptochlorogenic acid | 0.21 | 0.58 | 0.16 | 103.21 | 1.21 |
| Neochlorogenic acid | 0.24 | 0.21 | 0.12 | 98.63 | 0.78 |
| 4,5-*O*-dicaffeoyl quinic acid | 0.32 | 0.24 | 0.16 | 95.52 | 1.76 |
| 3,5-*O*-dicaffeoyl quinic acid | 0.29 | 0.11 | 0.14 | 99.79 | 1.87 |
| Morroniside | 0.83 | 0.58 | 0.44 | 99.23 | 2.89 |
| 3,4-*O*-dicaffeoyl quinic acid | 0.27 | 0.05 | 0.11 | 101.01 | 0.99 |
| Swertiamarin | 0.21 | 0.06 | 0.05 | 104.23 | 1.25 |
| Sweroside | 0.15 | 0.06 | 0.10 | 98.96 | 0.70 |
| Centauroside | 0.83 | 0.62 | 0.37 | 99.76 | 2.54 |
| Secoxyloganin | 0.19 | 0.16 | 0.09 | 103.52 | 0.79 |

**Supplementary Figures**


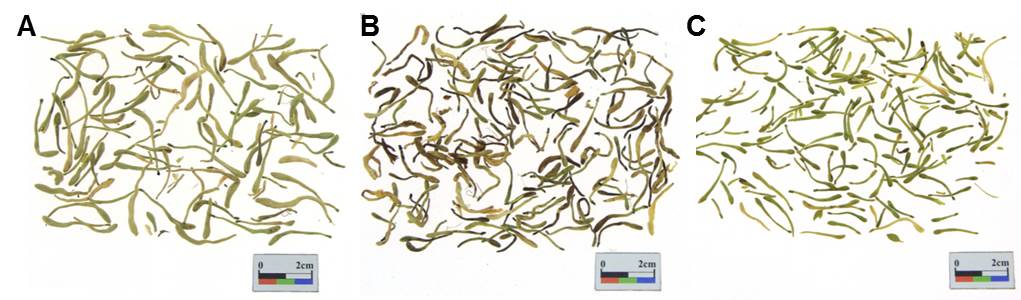


**Figure S1** Representative appearance of Lonicerae japonicae flos and Lonicerae flos. (A) cultivated Lonicerae japonicae flos; (B) wild Lonicerae japonicae flos; (C) Lonicerae flos.
